# An analysis of early insulin glargine added to metformin with or without sulfonylurea: impact on glycaemic control and hypoglycaemia

**DOI:** 10.1111/j.1463-1326.2011.01412.x

**Published:** 2011-09

**Authors:** V Fonseca, J Gill, R Zhou, J Leahy

**Affiliations:** 1 Department of Endocrinology, Tulane University Medical CenterNew Orleans, LA, USA; 2 sanofi-aventis U.S.Bridgewater, NJ, USA; 3 MedpaceCincinnati, OH, USA; 4 Division of Endocrinology, Diabetes and Metabolism, Department of Medicine, University of Vermont College of MedicineBurlington, VT, USA

**Keywords:** basal insulin therapy, insulin glargine, metformin, oral antidiabetic drugs, sulfonylurea, type 2 diabetes mellitus

## Abstract

**Aim:** To evaluate the benefits of initiating insulin at an earlier versus later treatment stage, and regimens with/without sulfonylurea (SU).

**Methods:** Pooled analysis of 11 prospective randomized clinical trials, including 2171 adults with uncontrolled type 2 diabetes initiating insulin glargine following a specific titration algorithm. Clinical outcomes were glycated haemoglobin A1c (HbA1c) reduction, per cent achieving HbA1c ≤ 7.0%, weight gain and hypoglycaemic events. Statistical analysis compared outcomes 24 weeks after basal insulin initiation in patients previously uncontrolled on 0/1 oral antidiabetic drug (OAD) versus 2 OADs, and in patients taking metformin (MET) or SU alone or in combination at baseline. A meta-analysis was also conducted.

**Results:** For the pooled analysis, patients on 0/1 OAD and those on MET monotherapy at baseline had the largest 24-week reductions in HbA1c following the addition of insulin glargine (∼0.44 U/kg). Of patients failing MET/SU monotherapy and MET + SU in combination, 68.1, 50.4 and 56.4% achieved HbA1c ≤ 7.0%, respectively (p = 0.0006). Weight gain was lowest when basal insulin was added to MET. Patients on 0/1 OAD at baseline had significantly less symptomatic hypoglycaemia when basal insulin was added than those on 2 OADs (p = 0.0007). Despite higher insulin doses, those taking MET alone had less hypoglycaemia than those taking SU or MET + SU. Results were confirmed in the meta-analysis.

**Conclusion:** Adding insulin glargine to MET monotherapy early in treatment may provide efficacy/safety benefits over regimens including SU. This may reflect treatment earlier in the disease and supports the inclusion of insulin as a second step in the American Diabetes Association/European Association for the Study of Diabetes treatment algorithm.

## Introduction

In 2009, the American Diabetes Association and the European Association for the Study of Diabetes (ADA/EASD) issued a consensus statement on the initiation and adjustment of therapy for type 2 diabetes [1]. This statement highlighted the critical importance of promptly achieving glycaemic control [i.e. glycated haemoglobin A1c (HbA1c) level of <7.0%], given the evidence that diagnosis and intervention early in the course of the disease process leads to lower HbA1c levels over time and reduces the risk of diabetes-related long-term complications [1,2]. With this goal in mind, the consensus algorithm adopted a tiered approach in which tier 1 interventions—well-validated core therapies—were deemed to ‘represent the best established and most effective and cost-effective therapeutic strategy for achieving the target glycaemic goals' [1]. These tier 1 interventions included lifestyle modification and metformin (MET) concomitantly initiated at step 1, the addition of either basal insulin or sulfonylurea (SU) therapy to MET at step 2 (if glycaemic goals are not achieved with MET alone), and the addition or intensification of insulin therapy as needed to attain glycaemic control at step 3 [1]. In routine clinical practice, however, insulin therapy is more often initiated after two or more oral antidiabetic drugs (OADs) have proven inadequate to achieve or maintain glycaemic control. The reasons for delaying insulin initiation are varied, but may include patient and physician perceptions that insulin therapy regimens are too complex, concerns about self-administering injections, or fears regarding side effects such as hypoglycaemia and weight gain [3,4].

For these reasons, we sought to use the extensive clinical trial database of insulin glargine to assess the observed clinical outcomes of earlier versus later basal insulin initiation on glycaemic control and safety after 24 weeks of treatment. We conducted a pooled analysis of randomized, controlled trials to compare clinical outcomes after initiating insulin glargine in patients with uncontrolled type 2 diabetes on 0 or 1 OAD versus 2 OADs at baseline and to determine if there are any differences between regimens containing MET alone, SU alone or MET + SU together. We also performed a meta-analysis to evaluate the robustness of the pooled analysis and to control for differences in sample size.

## Methods

### Study and Patient Selection Criteria for Pooled Analysis

In total, 63 randomized controlled trials evaluating insulin glargine in patients with type 2 diabetes have been conducted by sanofi-aventis or predecessor companies of sanofi-aventis. These studies were performed between 1997 and 2007, and individual patient data were available for inclusion in pooled analyses. Studies were deemed eligible for pooling if they met the following criteria:

- were phase 3 or later, prospective, randomized, controlled trials of ≥24 weeks' duration;
- enrolled adult patients with type 2 diabetes with inadequate glycaemic control;
- basal insulin was given once daily, with no concomitant prandial or bolus insulin administration;
- insulin glargine was initiated at 10 U/days and was administered according to predefined titration algorithms with frequent insulin dose adjustment (from every 1 to 3 days, to every week) to achieve fasting plasma glucose levels <100 mg/dl (<5.5 mmol/l); and
- studies were conducted according to Good Clinical Practice and in accordance with the Declaration of Helsinki.

Twelve studies met these eligibility criteria; however, one study discontinued thiazolidinediones abruptly at baseline and was not included in this analysis. Therefore, data from 11 studies were used in the pooled analysis (Table 1) [5–14] (Data on file, HOE-901-4021). In all studies, baseline OADs were to remain stable throughout the treatment period. Only data from patients in the insulin glargine arm of each of the 11 trials were examined for inclusion in this analysis (n = 2171). For patients in studies having treatment durations longer than 24 weeks, only data from the first 24 weeks were used.

**Table 1:** Clinical trials included in pooled and meta-analysis

Study reference (study no.)	Treatment/ comparator	Treatment duration (weeks)	N (total)	n (insulin glargine)
Gerstein et al.* [5] (3502)	Insulin glargine/OADs	26	405	197
Riddle et al.* [6] (4002)	Insulin glargine/NPH insulin	24	756	355
Standl et al. [7] (4009)	Insulin glargine (am)/insulin glargine (pm)	24	624	590
Rosenstock et al. [8] (4014)	Insulin glargine/rosiglitazone	24	219	104
Meneghini et al. [14] (4020)	Insulin glargine/pioglitazone	24/48	353	159
Data on file, HOE-901-4021 (4021)	Insulin glargine/insulin lispro 75/25	24	212	112
Janka et al. [9] (4027)	Insulin glargine/NPH insulin 70/30	28	371	175
Bretzel et al.* [10] (4040)	Insulin glargine/insulin lispro	44	415	198
Yki-Jarvinen et al.* [11] (4041)	Insulin glargine (group education)/insulin glargine (individual education)	24	121	119
Blickle et al. [12] (4042)	Insulin glargine/hygienic and dietary measures	40	215	101
Yki-Jarvinen et al.* [13] (6001)	Insulin glargine/NPH insulin	36	110	61
Pooled analysis	Insulin glargine	24	—	2171
Meta-analysis	Insulin glargine	24	—	930

OAD, oral antidiabetic drug.

* Studies included in the meta-analysis.

### Clinical Outcomes

Study endpoints for this analysis included week 24 HbA1c level and change from baseline, the percentage of patients reaching a target HbA1c level of ≤7.0%, change in body weight from baseline, insulin dose at endpoint and symptomatic and severe hypoglycaemic incidence and event rates during the treatment period.

### Statistical Analysis

HbA1c level and change from baseline to week 24 were analysed continuously with an analysis of covariance (ancova) model with baseline OAD use (OADs used up to the day before randomization) and study as factors and baseline HbA1c as a covariate. Week 24 HbA1c was dichotomized to ≤7.0 and >7.0%. The general association between the success of reaching target HbA1c and the use of OADs at baseline was assessed by a Cochran–Mantel–Haenszel (CMH) statistical test by controlling studies. Change from baseline to week 24 in body weight was analysed by an ancova model with baseline OAD classes and study as factors and baseline body weight as a covariate. The number and proportion of patients who had hypoglycaemia were analysed by the CMH test. Event rates per subject year were calculated and analysed continuously with an analysis of variance model, with study and baseline OAD classes as factors. Symptomatic hypoglycaemia was defined as all reported hypoglycaemia events with symptoms. Severe symptomatic hypoglycaemia was defined as all symptomatic hypoglycaemia requiring assistance with a blood glucose level of <36 mg/dl (<2 mmol/l; if available), and a prompt response to treatment with oral carbohydrates, intravenous glucose or glucagon.

### Meta-analysis Study Selection

**Figure 1:**
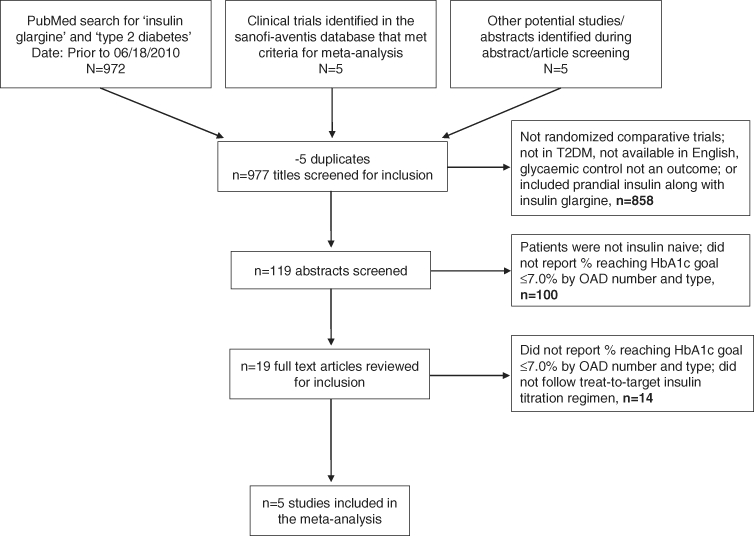
Flow of information through the systematic review.

We conducted a meta-analysis of studies to further explore the effects of the addition of basal insulin in participants uncontrolled on 0/1 versus 2 OADs and any significant differences when added to MET versus MET + SU or SU only. This analysis functioned as a sensitivity analysis to assess the robustness of the pooled analysis while controlling for effects such as sample size. Figure 1 shows the flow of information for the systematic review. To identify any additional studies similar to the trials in the pooled analysis for possible inclusion, we performed a thorough literature search of PubMed using the search terms ‘insulin glargine’ and ‘type 2 diabetes' for articles published before 18 June 2010. Titles were reviewed to identify randomized, controlled clinical trials in patients with type 2 diabetes treated with basal insulin glargine alone (no prandial insulin). Studies must have been available in English, and have glycaemic outcomes. Abstracts and full text articles were reviewed to determine if the study included patients who were insulin-naive and inadequately controlled on OADs prior to randomization to basal insulin therapy, if they reported the number of patients achieving glycaemic control (HbA1c ≤ 7.0%) at ≥24 weeks with 0/1 versus 2 OADs, MET versus MET + SU and SU versus MET + SU as outcomes, and used insulin titration algorithms. Of 977 articles initially identified for review, only 5 met the strict criteria for inclusion in this meta-analysis [5–14]. All five of these articles were included in the 11 studies that were included in the pooled analysis (Table 1). The other six studies, included in the pooled analysis, did not qualify because all patients were taking 1 OAD only or ≥2 OADs only at baseline.

### Meta-analysis Statistical Methodology

For this meta-analysis, the effect sizes were calculated as:

1. log odds ratio (OR) of glycaemic control (week 24 HbA1c ≤7.0%) for 0/1 OAD versus 2 OADs, as well as for MET versus MET + SU, SU versus MET + SU and MET versus SU and
2. log OR of experiencing a hypoglycaemic event during the 24 weeks for 0/1 OAD versus 2 OADs, as well as for MET versus MET + SU, SU versus MET + SU and MET versus SU.

For qualified studies, the effect size (log OR) was calculated together with the standard error. Heterogeneity of effect sizes was tested across trials with the Cochran *Q* test with I^2^ index [15]. A fixed-effects meta-analysis model was used, with the pooled effect for each grouping of trials weighted by the estimated inverse of the variance (1/s.e.^2^). Random-effects models also were used to estimate the pooled effect.

## Results

### Clinical Characteristics and Patient Demographics

Of 3801 patients, a total of 2171 in the 11 selected studies were treated with insulin glargine and therefore examined for inclusion in the pooled analysis. Before adding basal insulin, approximately 1.8% of patients were taking no OAD, 45.2% took 1 OAD and 52.2% took 2 OADs. Because only a very small sample of patients [17 (0.8%)] were taking three or more OADs at baseline, these 17 were excluded from the analysis. Nearly, half (49.9%) of all patients were taking MET + SU combination therapy before basal insulin treatment; 36.5% of patients took an SU only and 8.5% took a MET only. Participants not taking MET or SU were not included in OAD class analysis.

The demographics and baseline characteristics of the 2154 patients included in the final pooled analysis are shown in Table 2. Overall, the mean age of these patients was 58.6 years; 55.6% of patients were males, 88.3% were White and the mean number of years from diabetes diagnosis to screening was 8.9 years. The demographics and baseline characteristics for patients taking 0/1 OAD and 2 OADs at baseline were generally similar, although, as might be expected, those in the 2 OAD group had a longer duration of diabetes (9.3 vs. 8.4 years, p < 0.0001). Demographics and baseline characteristics for patients taking MET only, SU only or both at baseline were also generally similar, although the groups differed in age and duration of diabetes; patients taking MET alone were the youngest and had the shortest disease duration.

**Table 2:** Demographics and baseline characteristics by baseline OAD use in the pooled study and meta-analysis

		Baseline number of OADs		Type of OAD		
				
Characteristic*	Overall	0/1 OAD	2 OADs	MET	SU	MET + SU
Pooled study†						
N	2154	1020	1134	185	792	1084
Age (years)	58.6 (10.1)	59.5 (10.6)	57.9 (9.5)	52.5 (9.9)‡	61.2 (10.1)‡	57.9 (9.5)‡
Sex (male; %)	55.6	56.0	55.3	51.9	56.4	54.9
Race (White; %)	88.3	90.7	85.8	71.9	95.6	86.1
Region (USA; %)	31.3	21.2	40.4	58.9	12.8	38.8
History of diabetes (years)	8.9 (6.2)	8.4 (6.0)	9.3 (6.4)§	6.0 (4.1)‡	9.1 (6.2)‡	9.3 (6.4)‡
Baseline HbA1c (%)	8.77 (1.05)	8.87 (1.07)	8.68 (1.02)	9.08 (1.28)	8.84 (1.02)	8.67 (1.03)
Baseline FPG (mg/dl)	198.8 (52.3)	201.6 (51.6)	196.2 (52.7)	214.2 (60.2)	199.3 (49.4)	195.8 (52.9)
Baseline body weight (kg)	88.5 (17.7)	86.2 (17.4)	90.6 (17.7)	95.4 (19.8)	84.0 (16.3)	90.3 (17.5)
Meta-analysis‖						
N	928	271	657	97	135	609
Age (years)	57.1 (9.5)	56.8 (9.7)	57.3 (9.4)	53.8 (9.2)	58.8 (9.4)	57.2 (9.3)
Sex (male; %)	58.6	62.4	57.1	57.7	63.0	56.3
Race (White; %)	88.2	88.6	88.1	90.0	88.9	88.5
Region (USA; %)	32.2	21.0	36.8	21.6	25.2	34.0
History of diabetes (years)	8.3 (5.74)	6.6 (4.5)	9.0 (6.0)	5.8 (3.5)	7.5 (4.7)	9.1 (6.1)
Baseline HbA1c (%)	8.69 (1.04)	8.70 (1.11)	8.68 (1.00)	8.72 (1.20)	8.71 (1.08)	8.67 (1.01)
Baseline FPG (mg/dl)	199.7 (51.1)	195.4 (48.9)	201.5 (51.9)	198.2 (54.6)	194.7 (46.9)	201.3 (52.3)
Baseline body weight (kg)	90.8 (17.9)	89.9 (17.4)	91.2 (18.1)	94.9 (18.9)	88.0 (16.4)	90.7 (17.7)

FPG, fasting plasma glucose; MET, metformin; OAD, oral antidiabetic drug; SU, sulfonylurea.

* Data are mean (s.d.) unless otherwise specified.

† n = 39 were not on any OAD; n = 71 were on ‘other’.

‡ p < 0.05 for class comparison using an analysis of variance model with study and baseline OAD class as factors.

§ p < 0.05 versus 0/1 OAD group.

‖ n = 36 were not on any OAD; n = 51 were on ‘other’.

### Glycaemic Outcomes

**Figure 2:**
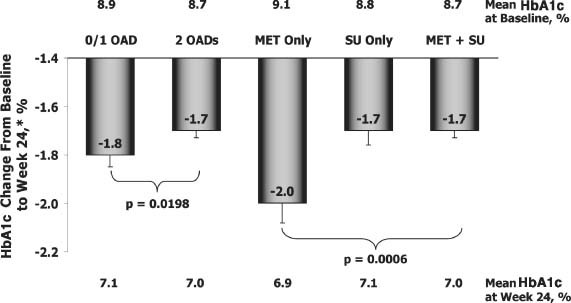
Change in HbA1c from baseline to week 24 by number and type of OAD at baseline (0/1 or 2; MET only, SU only, or both MET and SU). p Values were calculated from an analysis of variance model with study and baseline OAD class as factors and baseline HbA1c as a covariate. MET, metformin; OAD, oral antidiabetic drug; SU, sulfonylurea. *Least squares mean.

For the primary outcome of interest, percentage of patients reaching HbA1c ≤ 7.0% at week 24 after the addition of basal insulin, results were similar between the 0/1 OAD group and the 2 OAD group (54.7% vs. 56.7%, respectively, p = 0.0541). However, more patients in the MET-only group (68.1%) achieved HbA1c ≤ 7.0% than in the other groups (50.4 and 56.4% for SU only and combination groups, respectively; p = 0.0006 for comparison among all three groups, p = 0.0001 for MET vs. groups taking SUs). In addition, although there was no difference in mean endpoint HbA1c between groups, patients taking 0/1 OAD at baseline showed a significantly greater decrease in HbA1c from baseline to week 24 compared with the 2 OAD group (p = 0.0198; figure 2). Patients in the MET-only group experienced the largest mean improvement in HbA1c from baseline (−2.0% vs. −1.7% in each of the other two groups; p = 0.0006 for comparison among all three groups, p = 0.0001 for MET vs. groups taking SUs).

### Weight Change

**Figure 3:**
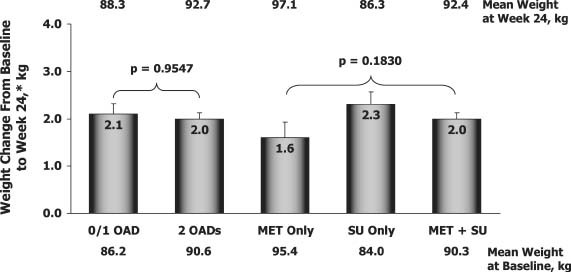
Change in body weight from baseline to week 24 by number and type of OAD at baseline (0/1 or 2; MET only, SU only, or both MET and SU). p Values were calculated from an analysis of variance model with study and baseline OAD use as factors and baseline body weight as a covariate. MET, metformin; OAD, oral antidiabetic drug; SU, sulfonylurea. *Least squares mean.

Weight gain from baseline to week 24 was not significantly different, based on either the number or type of OAD therapy at baseline (figure 3). However, the MET-only group had the numerically lowest weight gain over 24 weeks (1.6 vs. 2.3 kg in the SU-only group and 2.0 kg in the combination group, p = 0.1830) after basal insulin initiation.

### Insulin Dose

The stable weight-based insulin doses for patients on 0/1 OAD and on 2 OADs were similar (0.45 vs. 0.43 U/kg); however, the mean insulin dose per kilogram in patients on MET only was higher than that for patients on SU only or on SU + MET combination therapy (0.54 vs. 0.43 vs. 0.43 U/kg, respectively). As would be expected, insulin dose was positively correlated with weight gain overall (Pearson r^2^ = 0.3167, p < 0.0001). In the MET-only group, however, weight gain was statistically similar and numerically lower than in the other groups, despite the higher insulin dose.

### Hypoglycaemia

As shown in Table 3, symptomatic, confirmed symptomatic with glucose <50 mg/dl and severe hypoglycaemia incidence and rates were low overall. Patients in the 0/1 OAD group had lower incidence and rates of symptomatic and confirmed (blood glucose <50 mg/dl) hypoglycaemia versus those taking 2 OADs, and those taking MET alone had lower incidence and rates than those taking SU alone or in combination, with the highest incidence in the MET + SU combination group. The incidence of severe symptomatic hypoglycaemia followed the same overall pattern, but did not reach statistical significance.

**Table 3:** Incidence and event rate of symptomatic, confirmed symptomatic with blood glucose <50 mg/dl and severe hypoglycaemia during 24 weeks

Baseline OAD number/class	Incidence, n/N (%)	p*	Event rate per subject-year, least squares mean (s.e.)	p*
Symptomatic hypoglycaemia				
0/1 OAD	424/1020 (41.6)	0.0007	4.05 (0.71)	0.0009
2 OADs	713/1134 (62.9)		7.18 (0.43)	
MET only	51/185 (27.6)	<0.0001	1.81 (1.06)	<0.0001
SU only	352/792 (44.4)		4.88 (0.90)	
MET + SU	687/1084 (63.4)		7.30 (0.43)	
Confirmed symptomatic hypoglycaemia with blood glucose <50 mg/dl†				
0/1 OAD	175/1013 (17.3)	0.0122	0.99 (0.22)	0.0643
2 OADs	375/1080 (34.7)		1.53 (0.13)	
MET only	16/178 (9.0)	0.0006	0.67 (0.34)	0.0576
SU only	149/792 (18.8)		1.05 (0.27)	
MET + SU	365/1030 (35.4)		1.56 (0.14)	
Severe symptomatic hypoglycaemia†				
0/1 OAD	12/1013 (1.2)	0.6444	0.01 (0.05)	0.4416
2 OADs	14/1080 (1.3)		0.06 (0.03)	
MET only	2/178 (1.1)	0.6329	0.00 (0.07)	0.7231
SU only	10/792 (1.3)		0.02 (0.06)	
MET + SU	14/1030 (1.4)		0.06 (0.03)	

Event rate per subject-year = 365.25 × number of events/exposure (days). MET, metformin; OAD, oral antidiabetic drug; s.e., standard error; SU, sulfonylurea.

* p Values for categorical variables were from Cochran–Mantel–Haenszel statistics, which test general association between hypoglycaemia events and baseline OAD status. p Values for continuous variables were from analysis of variance models, including study and baseline OAD status as factors.

† Study 6001 was excluded because no detailed information of hypoglycaemia was collected.

### Meta-analysis

As described in the section Methods, 5 of the 11 sanofi-aventis trials from the pooled analysis met the criteria for inclusion in a meta-analysis, and no additional eligible studies were found based on a systematic literature review (figure 1 and Table 1). The studies included in this analysis included 928 participants who were administered insulin glargine in addition to 0/1 OAD (n = 271) or 2 OADs (n = 657); and with MET only (n = 97), SU only (n = 135), or MET + SU (n = 609). Baseline demographics and clinical characteristics for this analysis population are shown in Table 2.

**Figure 4:**
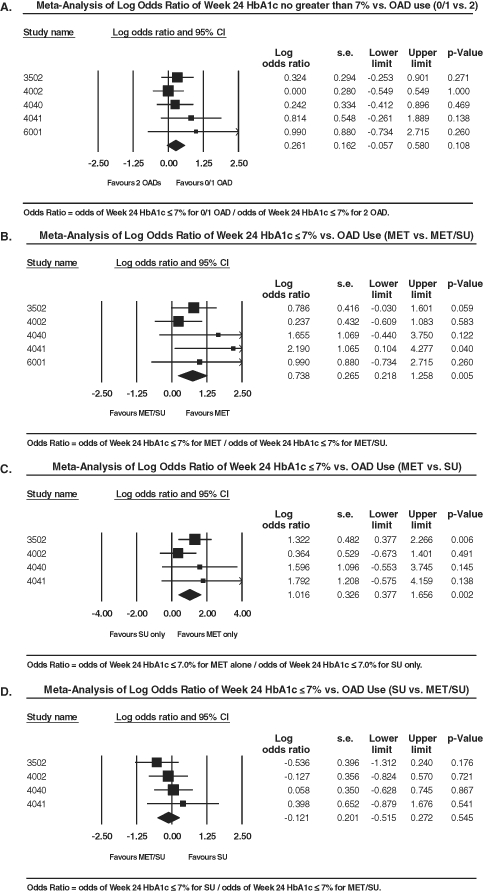
Meta-analysis results of log odds ratios for achieving HbA1c ≤7.0% at week 24 with baseline OAD use of (A) 0/1 versus 2 OADs, (B) MET only versus MET + SU, (C) MET monotherapy versus SU monotherapy and (D) SU monotherapy versus MET + SU. Because all patients in study 6001 were taking either MET monotherapy or combination therapy with MET + SU, this study was not included in comparisons with SU monotherapy (panels C and D). CI, confidence interval; MET, metformin; OAD, oral antidiabetic drug; SU, sulfonylurea.

The results for the log OR of achieving glycaemic control (HbA1c ≤7.0%) at 24 weeks with each regimen are shown in figure 4. For each of the comparisons, the fixed- and random-effects models agreed, therefore, results from the fixed-effects model are presented. In addition, all comparisons met the assumption for homogeneity with the Cochran *Q*, p > 0.05; I^2^ = 0 except where noted. The log OR of reaching HbA1c target slightly, but not significantly, favoured the addition of basal insulin to baseline therapy with 0/1 OAD versus 2 OADs [OR (95% confidence interval): 0.261 (−0.057 to 0.580), p = 0.108; figure 4A], Cochran *Q* = 2.620, p = 0.623. When analysed by the type of OAD, the estimated OR of achieving HbA1c target was significantly greater for the addition of basal insulin to baseline MET monotherapy versus MET + SU combination [0.738 (0.218 to 1.258), p = 0.005] or SU monotherapy [1.016 (0.377 to 1.656), p = 0.002]. There was no significant difference between baseline SU monotherapy and MET + SU combination therapy (figure 4B–D), Cochran *Q*, p > 0.40 for all and I^2^ = 0.956 for MET versus MET + SU.

**Figure 5:**
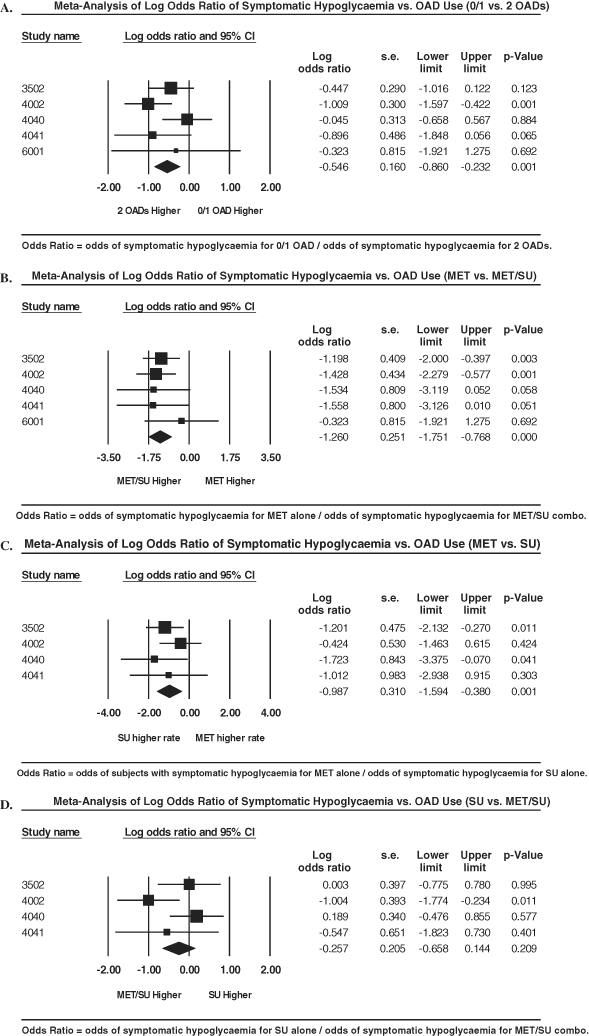
Meta-analysis results of log odds ratios for experiencing symptomatic hypoglycaemia with baseline OAD use of (A) 0/1 versus 2 OADs, (B) MET monotherapy versus MET + SU, (C) MET monotherapy versus SU monotherapy and (D) SU monotherapy versus MET + SU. Because all patients in study 6001 were taking either MET monotherapy or combination therapy with MET + SU, this study was not included in comparisons with SU monotherapy (panels C and D). CI, confidence interval; MET, metformin; OAD, oral antidiabetic drug; SU, sulfonylurea.

Results from the meta-analysis for frequency of symptomatic hypoglycaemia are shown in figure 5. Adding basal insulin to 0/1 OAD was associated with a significantly lower risk of hypoglycaemia versus adding basal insulin to 2 OADs [−0.546 (−0.860 to −0.232), p = 0.001; Cochran *Q* = 5.660, p = 0.226 and I^2^ = 29.332, figure 5A). Likewise, risk of hypoglycaemia was lower with baseline MET monotherapy versus MET + SU combination therapy [−1.260 (−1.751 to −0.768), p < 0.001] or SU monotherapy [−0.987 (−1.594 to −0.380), p = 0.001]. There was no significant difference between baseline therapy with SU alone and MET + SU combination therapy (Cochran *Q*, p > 0.10 for all and I^2^ = 49.72 for SU vs. MET + SU, figure 5B–D).

## Discussion

In this large, pooled analysis of prospective, randomized, controlled clinical trials in patients with type 2 diabetes, more than half of the patients previously uncontrolled on 0, 1 or 2 OADs with baseline HbA1c of 8.7–9.1% achieved a target HbA1c ≤7.0% after 24 weeks of treatment with the addition of basal insulin, while continuing the oral agent(s). Patients taking MET alone before the initiation of basal insulin had the greatest HbA1c reductions and the largest proportion of patients achieving glycaemic goal at week 24 compared with patients taking an SU alone or in combination with MET. Hypoglycaemia rates were low overall. Patients on MET only at baseline also had the lowest rate of symptomatic and severe hypoglycaemia and the lowest weight gain after 24 weeks of treatment with insulin glargine in spite of a higher insulin dose on average. The results from this pooled analysis were supported by a meta-analysis of five eligible studies from the trial cohort in which the OR of achieving glycaemic goals at week 24 was significantly higher and risk of symptomatic hypoglycaemia was significantly lower for patients taking MET alone before initiation of basal insulin compared with those taking an SU alone or in combination with MET.

These data illustrate that adding basal insulin in patients whose hyperglycaemia remained uncontrolled on 0/1 OAD (and particularly to MET monotherapy) resulted in greater HbA1c reductions and lower rates of hypoglycaemia after 24 weeks than adding basal insulin to 2 OADs. These findings provide evidence-based clinical trial support for the success of the tier 1 consensus strategy from the ADA/EASD [1], recommending the addition of basal insulin (or SU therapy) to MET monotherapy plus lifestyle intervention at step 2 (i.e. if MET plus lifestyle intervention alone is unable to achieve glycaemic control).

Moreover, these preliminary observations suggest the value of adding basal insulin to MET monotherapy early in the clinical management of type 2 diabetes as a viable option for achieving glycaemic control, because patients on 0/1 OAD in the trials analysed here were at an earlier stage of their type 2 diabetes. These findings have notable clinical implications, given that a common strategy in real-world clinical practice is to delay insulin initiation in patients with type 2 diabetes for as long as possible. Although patient concerns and other barriers to the implementation of insulin therapy are important to consider, clinical evidence continues to accumulate highlighting the urgent need to overcome these barriers to maximize patient benefit by initiating insulin therapy earlier in the course of the disease. This is certainly possible in today's treatment landscape, with the availability of long-acting basal insulin formulations that can be administered once daily and titrated using simple algorithms. In addition, insulin pen delivery devices make injections easier and more convenient for patients. Keys to implementing this strategy include increasing physician awareness of the importance of earlier insulin initiation and patient education regarding the need for improved glycaemic control if targets are not being met.

Strengths of this analysis are its large size (including data from more than 2000 patients treated with basal insulin in 11 clinical trials evaluated in the pooled analysis), wide geographic variation and reliance on data from prospective, controlled trials. In addition, although the sample sizes for each of the OAD treatment groups differed substantially, these differences were eliminated when evaluating effects with a meta-analysis. Specifically, the number of patients on MET monotherapy prior to randomization was much smaller than those in the other two groups, and the results from the meta-analysis were consistent with the pooled analysis in that adding basal insulin to MET monotherapy provided beneficial glycaemic control.

One major limitation of this analysis was that only studies of insulin glargine were evaluated in both the pooled and meta-analyses; thus, applicability to other basal insulin formulations (e.g. NPH insulin and insulin detemir) is unknown. It should also be noted that our analyses focus on the number and type of OADs that were used at initiation of basal insulin glargine. The OADs were to be maintained over 24 weeks of treatment in all studies; however, we did not assess if any changes did occur in OADs during the course of this study. Therefore, these results provide important information to be considered by clinicians specific to deciding when to initiate insulin therapy in people failing OAD treatment. The best approach for optimizing and maintaining glycaemic control over time should be individualized for each patient. Furthermore, this study addresses outcomes following 24 weeks of treatment; the impact on longer term outcomes such as clinical complications or death was not assessed. Another limitation of this analysis is that although statistical pooling of data in pooled- and meta-analyses increases statistical power and may result in more precise estimates of therapeutic effect, these are hypotheses generating analyses; additional randomized controlled clinical trials are needed to draw reliable conclusions.

In conclusion, patients adding basal insulin glargine to 0/1 OAD at baseline, who are in earlier stages of type 2 diabetes, showed a greater reduction in HbA1c with lower risk of hypoglycaemia than those failing 2 OADs at baseline. In particular, adding insulin glargine to MET monotherapy was well tolerated and resulted in a significant proportion of patients achieving the glycaemic goal of HbA1c ≤7.0% with a low risk of hypoglycaemia and weight gain, in spite of a higher insulin dose used on average. These findings suggest that some patients may benefit from the initiation of basal insulin earlier in the management of type 2 diabetes than often occurs in this clinical practice, which further corroborates the guidance outlined for the clinical management of type 2 diabetes by the ADA and EASD.
